# Two-Hour Post-dose (C2)-Monitored Cyclosporine Microemulsion as a Practical Alternative to Voclosporin in Mixed Class IV/V Lupus Nephritis: A Case Report

**DOI:** 10.7759/cureus.102585

**Published:** 2026-01-29

**Authors:** Divya Akella, Virin Ramoutar

**Affiliations:** 1 Division of Nephrology, Medical College of Georgia, Augusta University, Augusta, USA

**Keywords:** c2 monitoring, calcineurin inhibitors, calcineurin inhibitors (cni), cost effective, cyclosporine microemulsion csa-me, lupus nephritis (ln), mixed class iv + v ln, non depletive immunosuppression, precision dosing, proteinuria remission

## Abstract

Lupus nephritis (LN) is a major cause of chronic kidney disease and progression to end-stage kidney disease in patients with systemic lupus erythematosus. Calcineurin inhibitor-based therapy is used in LN management because of its combined systemic immunomodulatory effects on T-cell activation and direct podocyte-stabilizing actions, leading to reduction in proteinuria. We report a patient with mixed class IV and V LN who achieved partial remission with voclosporin but was unable to continue therapy. Transition to cyclosporine microemulsion (CsA-ME) guided by two-hour post-dose (C2) monitoring was associated with complete proteinuric remission and stable kidney function during follow-up. This case demonstrates that individualized C2-guided CsA-ME dosing was associated with marked proteinuria reduction and preservation of kidney function. The observed response suggests pharmacodynamic effects that may overlap with those reported for voclosporin, within a fully oral, non-depletive regimen selected to mitigate treatment-related toxicity from cytotoxic therapy after shared decision-making with the patient.

## Introduction

Lupus nephritis (LN) is a major cause of morbidity and progression to kidney failure in systemic lupus erythematosus, particularly in proliferative (class III/IV) and membranous (class V) disease, both of which are associated with relapse risk and significant long-term kidney morbidity [1]. Standard induction regimens rely on mycophenolate mofetil or cyclophosphamide in combination with glucocorticoids; however, cytotoxic therapy is associated with infertility, infection, hypogammaglobulinemia, and cumulative organ toxicity [1]. Calcineurin inhibitors (CNIs) such as voclosporin, cyclosporine, and tacrolimus have therefore gained prominence as adjuncts or alternatives based on clinical trial data and mechanistic evidence demonstrating both systemic immunomodulatory effects through inhibition of T-cell activation and direct podocyte-stabilizing actions that accelerate proteinuria reduction [1,2].

Voclosporin provides relatively predictable pharmacokinetics without the need for therapeutic drug monitoring and improves renal response rates when added to mycophenolate and glucocorticoids, as demonstrated in the AURORA-1 and AURORA-2 trials [3,4]. Although cost-effective in modeling studies, high absolute cost and variable insurance coverage limit real-world access [5]. Cyclosporine microemulsion (CsA-ME), when guided by two-hour post-dose (C2) monitoring, offers a pharmacokinetically rational strategy to target antiproteinuric exposure at substantially lower cost [6-9]. C2 levels more closely reflect cyclosporine area under the concentration-time curve than trough measurements and correlate more strongly with both efficacy and toxicity [6-9]. Cyclosporine differs pharmacologically from voclosporin in its greater interpatient variability in absorption and metabolism, necessitating therapeutic drug monitoring, as supported by transplant literature demonstrating superior correlation of C2 levels with cyclosporine exposure and adverse effects compared with trough concentrations [9,10]. Two-hour post-dose (C2) monitoring therefore enables individualized dosing to balance efficacy and toxicity.

We describe a patient with severe class IV + V LN who demonstrated an initial response to voclosporin but required transition to CsA-ME because of insurance-related loss of access. C2-guided CsA-ME dosing was associated with sustained proteinuric remission using a fully oral, non-depletive regimen selected to mitigate treatment-related toxicity from cytotoxic therapy after shared decision-making with the patient.

## Case presentation

A 43-year-old African American man with hypertension, prediabetes, chronic kidney disease stage 2, and nephrotic-range proteinuria presented with progressive generalized edema. Serologic evaluation revealed antinuclear antibody positivity (1:320, speckled pattern), anti-Smith and anti-U1 RNP antibodies, and markedly reduced complement levels.

**Figure 1:**
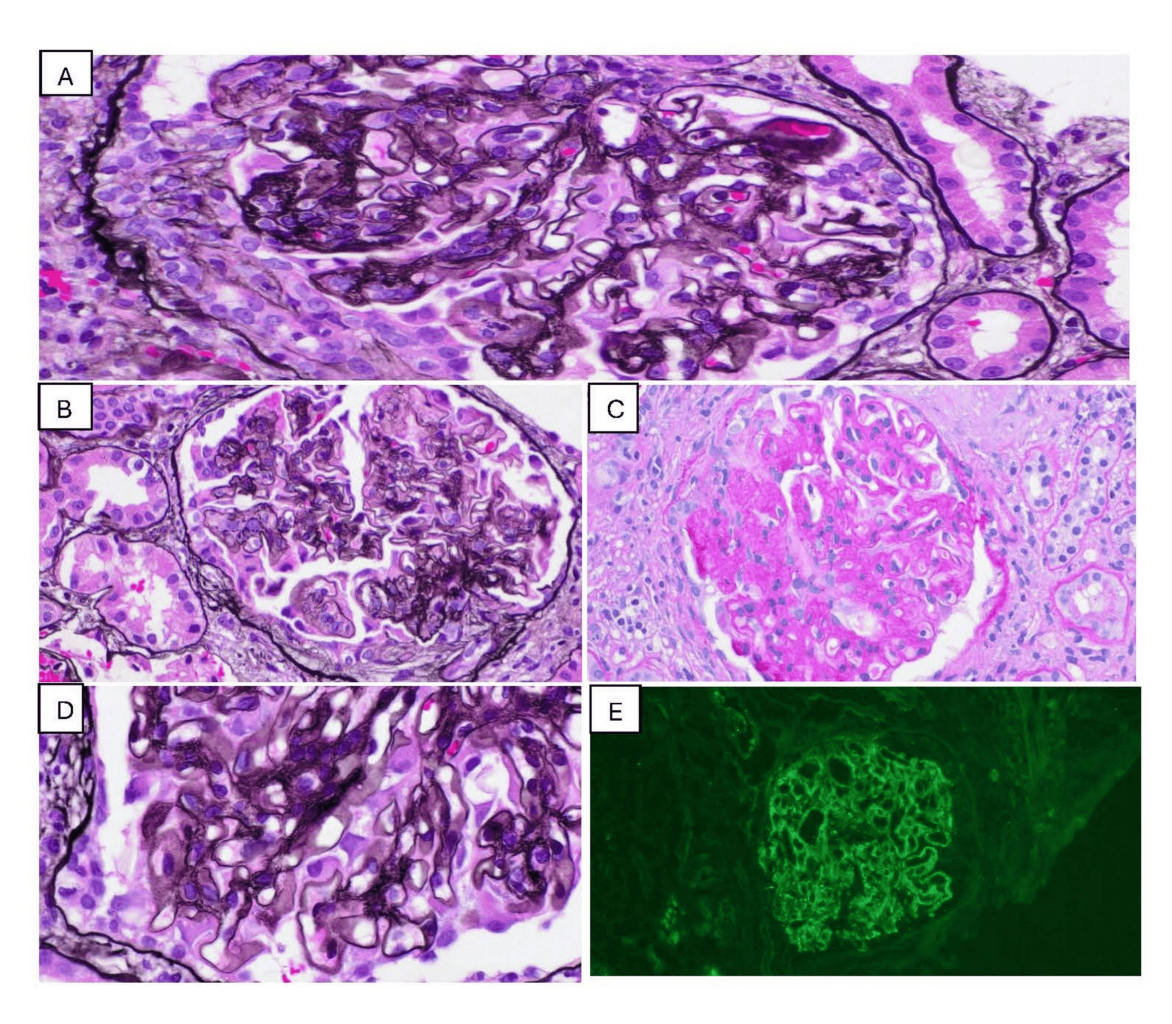
Renal biopsy findings consistent with mixed class IV + V lupus nephritis. A. Light microscopy demonstrating a fibrocellular crescent. B. Endocapillary hypercellularity with active proliferative lesions. C. Segmental glomerulosclerosis. D. Silver stain showing glomerular basement membrane spikes and holes, consistent with membranous features. E. Immunofluorescence demonstrating C3-dominant immune complex deposition (full-house pattern).

A kidney biopsy performed in December 2024 demonstrated mixed class IV + V LN, with mesangial and endocapillary hypercellularity, full-house immune complex deposition, and five fibrocellular crescents among 35 sampled glomeruli (14.3%). Chronicity was limited, with <10% interstitial fibrosis and tubular atrophy (Figure 1).

The modest crescent burden and minimal chronic damage indicated preserved renal reserve and provided a therapeutic window to pursue a non-cytotoxic induction strategy.

Although cyclophosphamide and biologic therapy (e.g., rituximab) were considered and available, a shared decision-making process favored a fully oral, non-depletive CNI-based regimen to mitigate treatment-related toxicity while targeting rapid proteinuria reduction. Treatment was initiated in January 2025 with losartan 100 mg daily, empagliflozin 10 mg daily, mycophenolate mofetil 1.5 g twice daily, hydroxychloroquine 400 mg daily, and glucocorticoids. Prednisone was started at 60 mg daily and tapered by 10 mg every two weeks, reaching 20 mg daily by April 2025 and fully discontinued by September 2025.

Given persistent nephrotic-range proteinuria (urine protein-creatinine ratio (UPCR) 4.6 g/g), voclosporin 23.7 mg twice daily was added on February 15, 2025. Despite therapy, proteinuria remained nephrotic by April 15, 2025 (UPCR 4.9 g/g), with stable kidney function. Voclosporin was discontinued after approximately two months of treatment (February 15 to April 15, 2025) because of insurance-related coverage denial.

Transition to cyclosporine microemulsion

Cyclosporine microemulsion (CsA-ME) was initiated on April 15, 2025, at 250 mg twice daily, targeting a two-hour post-dose (C2) concentration of 600-800 ng/mL based on established transplant therapeutic drug monitoring data. Early C2 levels were supratherapeutic (>1500 ng/mL), prompting stepwise dose reductions to 150 mg twice daily and subsequently to 50 mg twice daily as part of initial exposure correction. Thereafter, CsA-ME dosing was dynamically titrated using serial C2 monitoring, with ongoing dose adjustments made to maintain concentrations within the target range of 600-800 ng/mL throughout therapy, consistent with transplant-based exposure targets.

C2 concentrations were measured using a standardized whole-blood immunoassay routinely employed for transplant therapeutic drug monitoring.

Therapeutic response and safety monitoring

Over five months of C2-guided CsA-ME therapy (April 15 to September 15, 2025), in combination with continued mycophenolate mofetil (1.5 g twice daily), hydroxychloroquine (400 mg daily), losartan (100 mg daily), empagliflozin (10 mg daily), and a structured glucocorticoid taper, the UPCR declined from 4.9 g/g to 0.47 g/g, accompanied by normalization of complement levels (Table 1).

**Table 1:** Therapeutic response. Trends in urine protein-to-creatinine ratio (UPCR), complement C3, and complement C4 from January 2025 to September 2025 during background antiproteinuric therapy, including voclosporin (February 15-April 15, 2025) followed by C2-guided cyclosporine microemulsion initiated on April 15, 2025, and continued through September 15, 2025.

Date	UPCR (mg/g) (ref: 22-128)	C3 (mg/dL) (ref: 90-170)	C4 (mg/dL) (ref: 19-52)
January 2025	13231	65	9
February 2025	4656	121	38
April 2025	4957	112	28
August 2025	512	130	36
September 2025	466	124	35

Serum creatinine remained stable at approximately 1.3 mg/dL throughout follow-up. The patient was monitored for cyclosporine-associated adverse effects, including blood pressure, serum magnesium, and blood glucose levels; all parameters remained stable during the observation period.

## Discussion

The 2024 Kidney Disease: Improving Global Outcomes (KDIGO) LN guidelines recognize CNIs as a therapeutic option in selected patients, particularly when used in combination with mycophenolate mofetil and glucocorticoids, while emphasizing individualized treatment selection and careful monitoring [1]. This guidance reflects evidence that CNI-containing regimens can accelerate proteinuria reduction in some patients, while also acknowledging uncertainty regarding durability of response and long-term renal safety.

From a systemic immunologic perspective, calcineurin inhibition suppresses nuclear factor of activated T cells (NFAT) signaling, resulting in reduced IL-2 transcription and downstream T-cell activation implicated in systemic lupus erythematosus immune dysregulation [2]. Clinically, voclosporin added to background mycophenolate and glucocorticoids improves short-term renal response rates and accelerates proteinuria reduction, as demonstrated in the AURORA-1 trial and sustained in its extension study, AURORA-2 [3,4]. Although voclosporin may be cost-effective under selected modeled scenarios, its high acquisition cost and inconsistent insurance coverage limit real-world accessibility, necessitating alternative strategies in some patients [5].

In addition to systemic immunomodulation, CNIs exert direct podocyte-specific effects that contribute to their antiproteinuric action. Cyclosporine stabilizes the podocyte actin cytoskeleton by preventing calcineurin-dependent dephosphorylation and cathepsin L-mediated degradation of synaptopodin [6]. Calcineurin-NFAT signaling also promotes maladaptive upregulation of transient receptor potential canonical 6 (TRPC6) channels; angiotensin II activates this pathway, increasing TRPC6 expression and promoting injurious calcium influx in podocytes [7]. TRPC6 has emerged as an important determinant of podocyte vulnerability in proteinuric kidney diseases [8]. Together, these mechanisms provide a biologically plausible explanation for the rapid reductions in proteinuria observed with CNI-based regimens, independent of slower systemic immunologic effects.

In the present case, voclosporin could not be continued because of insurance-related loss of access. Transition to CsA-ME therefore required individualized, exposure-guided dose adjustment. Two-hour post-dose (C2) monitoring more closely reflects cyclosporine area under the concentration-time curve than trough (C0) levels and correlates more strongly with both efficacy and toxicity in transplant populations [9-11]. Maintenance renal transplant studies have demonstrated that C2 targets in the range of 600-800 ng/mL provide adequate immunosuppression with acceptable safety profiles beyond the early post-transplant period. In a prospective cohort of stable kidney transplant recipients, Marcén R et al. reported favorable long-term outcomes in patients maintained within this C2 range, supporting its use as a pragmatic exposure target in maintenance settings [10]. Accordingly, CsA-ME dosing was dynamically adjusted using serial C2 measurements, with the objective of maintaining concentrations within this target range. In this patient, transplant-based starting doses resulted in supratherapeutic exposure, highlighting substantial interindividual pharmacokinetic variability and underscoring the importance of therapeutic drug monitoring rather than fixed, weight-based dosing.

Evidence supporting CNI use in LN remains heterogeneous. While randomized trials demonstrate improved short-term renal response with CNI-containing regimens, concerns persist regarding relapse after withdrawal, durability of response, and chronic nephrotoxicity with prolonged exposure [1]. Data from non-voclosporin CNI trials further inform this context; in a randomized study, multitarget therapy with tacrolimus, mycophenolate, and glucocorticoids achieved higher short-term remission rates than cyclophosphamide-based induction [12]. However, these findings do not resolve uncertainty regarding long-term renal outcomes or optimal patient selection.

Avoidance of cytotoxic and B-cell-depleting therapies was clinically meaningful for this patient. Cyclophosphamide remains a guideline-supported induction option in LN [1] but is associated with infertility, marrow suppression, urothelial toxicity, and cumulative malignancy risk, with infertility risk increasing with cumulative exposure [13]. Rituximab may be effective in refractory disease but can result in prolonged B-cell depletion and secondary hypogammaglobulinemia requiring immunoglobulin replacement [14]. Expert commentary emphasizes the importance of carefully weighing these risks in contemporary LN management [15]. In this context, a fully oral, non-depletive regimen was favored.

Cyclosporine was selected over tacrolimus in this case for pragmatic considerations, including the availability of validated C2 monitoring strategies, broad availability and lower cost of generic CsA-ME formulations, clinician familiarity with its pharmacokinetics, and integration of chronic kidney disease and transplant clinic infrastructure allowing streamlined exposure-guided dose adjustment [16]. These factors were particularly relevant in the setting of insurance-driven loss of access to voclosporin.

An additional theoretical advantage of C2-guided cyclosporine therapy relates to mitigation of calcineurin-associated nephrotoxicity. Cyclosporine generates multiple metabolites that variably contribute to vasoconstriction and tubular injury. Although voclosporin was structurally modified to reduce metabolite generation and pharmacokinetic variability, long-term renal safety data beyond approximately 18-24 months remain limited, a time frame when chronic calcineurin nephrotoxicity often becomes clinically apparent [4,16]. Precision-based exposure targeting may therefore represent a strategy to minimize cumulative toxic burden, although this hypothesis warrants formal evaluation.

Limitations

This report describes a single patient and is therefore not generalizable. Follow-up after transition to CsA-ME was short term, precluding assessment of long-term renal outcomes, relapse risk, or chronic CNI nephrotoxicity. Cyclosporine was administered in combination with mycophenolate and glucocorticoids, preventing isolation of its independent effect. Accordingly, this case should be viewed as hypothesis-generating and illustrative of feasibility rather than comparative efficacy.

## Conclusions

In this patient with mixed class IV and V lupus nephritis, C2-guided cyclosporine microemulsion therapy, in combination with mycophenolate, tapering glucocorticoids, angiotensin receptor blockade, and SGLT2 inhibition, was associated with complete proteinuric remission, normalization of complement levels, and preservation of kidney function. This fully oral, non-depletive regimen avoided exposure to cytotoxic and B-cell-depleting therapies. While the observed response is biologically plausible based on established calcineurin-mediated immunologic and podocyte-stabilizing mechanisms, conclusions regarding equivalence or generalizability cannot be drawn from a single case. C2-guided cyclosporine may represent a pragmatic, individualized alternative in selected patients when access to voclosporin is limited, warranting further systematic evaluation.
